# Formation of Thin, Isoporous Block Copolymer Membranes by an Upscalable Profile Roller Coating Process—A Promising Way to Save Block Copolymer

**DOI:** 10.3390/membranes8030057

**Published:** 2018-08-06

**Authors:** Thomas Bucher, Volkan Filiz, Clarissa Abetz, Volker Abetz

**Affiliations:** 1 Helmholtz-Zentrum Geesthacht, Institute of Polymer Research, Max-Planck-Str. 1, 21502 Geesthacht, Germany; thomas.bucher@hzg.de (T.B.); volkan.filiz@hzg.de (V.F.); clarissa.abetz@hzg.de (C.A.); 2 Institute of Physical Chemistry, University of Hamburg, Martin-Luther-King-Platz 6, 20146 Hamburg, Germany

**Keywords:** roller coating, self-assembly, block copolymer, isoporous membrane, pH responsiveness, thin layer, integral-asymmetric membrane, upscaling, ultrafiltration, film homogeneity

## Abstract

In this work we present a method to manufacture flat sheet membranes with a thin isoporous block copolymer (BCP) layer (thickness <3 µm) by profile roller coating (breadth: 30 cm) on top of a porous support membrane. Highly diluted BCP-solutions were used for this coating process. While we cast membranes with dimensions of 30 cm × 50 cm in this work, the procedure can easily be extended to endless dimensions in this roll to roll (R2R) process. The method offers the possibility to save >95% of BCP raw material compared to common doctor blade casting, by strongly decreasing the layer thickness to below 3 µm in combination with a highly open substructure. Additionally, we report a straightforward method to investigate the influence of the solvent evaporation time between coating and precipitation (phase inversion) on the membrane morphology using one sample only, which also ensures that all other influencing parameters remain constant.

## 1. Introduction

Membrane based separation techniques are used in various industries as water and wastewater treatment [1], gas separation, separation of chemicals and petrochemicals [2], haemodialysis [3] or in clinical sensors [4], in juice production [5], wine filtration [6,7,8], separation of biomolecules [9] or drug delivery [10].

Isoporous membranes have been traditionally prepared via ion-track etching [11,12] and inorganic aluminium anodic oxidation (AAO) [13]. While polycarbonate track-etched (PCTE) films suffer from their low pore density, AAO membranes show a high pore density with close hexagonal pore alignment in a range of 50–500 nm interpore distance that comes along with the brittle property of these films. First isoporous membranes from self-assembling block copolymers were prepared as thin layers on a solid substrate from solution and then transferred to a porous support [14]. A well-known process to generate polymer membranes with a thin selective layer on top of a more open porous spongy substructure from the same material is the non-solvent induced phase separation (NIPS), also known as “phase inversion”. This leads to mechanically robust membranes with both good selectivity and high permeance. Applying NIPS to self-assembling block copolymers (so-called SNIPS) can lead to so called integral isoporous membranes if the right conditions are found. This was shown for the first time for block copolymers (BCPs) like polystyrene-*b*-poly(4-vinylpyridine) (PS-*b*-P4VP) [15]. This leads to membranes with a highly ordered surface morphology, small pore size distribution and high porosity [16]. Specific properties of the pore forming block itself can induce responsive behaviour towards external stimuli like changing pH or even temperature after coating with polydopamine as an interlayer which is further modified with a temperature responsive polymer such as poly(*N*-isopropylacrylamide) (pNIPAM) [17,18]. Later on, suitable conditions for SNIPS were also found for a still increasing number of other block copolymers, namely poly(*tert*-butylstyrene)-*b*-poly(4-vinylpyridine) (PtBu-*b*-P4VP) [19], polystyrene-*b*-poly(solketal methacrylate) (PS-*b*-PSMA) [20], polystyrene-*b*-poly(ethylene oxide) (PS-*b*-PEO) [21], polystyrene-*b*-poly(methyl methacrylate) (PS-*b*-PMMA) [22], polystyrene-*b*-poly(2-hydroxyethyl methacrylate) (PS-*b*-PHEMA) [23] or even triblock terpolymers like polystyrene-*b*-poly(2-vinylpyridine)-*b*-poly(ethylene oxide) (PS-*b*-P2VP-*b*-PEO) [24], polyisoprene-*b*-polystyrene-*b*-poly(4-vinylpyridine) (PI-*b*-PS-*b*-P4VP) [25,26] or polyisoprene-*b*-polystyrene-*b*-poly(*N*,*N*-dimethylacrylamide) (PI-*b*-PS-*b*-PDMA) [27].

Besides expanding the number of block copolymer membranes obtained by SNIPS, many efforts have been made to understand the BCP membrane formation mechanism. Investigations were focused on the BCP structure formation in dependence of solvent selectivity [28], solvent composition [29], influence of additives helping to assemble the pore forming blocks by hydrogen bonds [30,31,32] or complex formation [33,34], the influence of the casting conditions like solvent evaporation time [35], temperature or humidity [36,37]. The tailored pore size of BCP membranes can be tuned by changing the ratio of both blocks, the total molecular weight [38] or blending [39]. The cast films are in a mobile, non-equilibrated state before precipitation. These wet non-equilibrated films get fixed at the moment of phase inversion, which is induced usually by the typical non-solvent water. Hexagonally or square-like packed cylinders form a 10 up to ca. 500 nm thick selective top layer [37] and merge into a much thicker sponge-like substructure of several tens of µm, which physically supports the thin but sensitive top layer and which is in the case of flat sheet membranes usually additionally supported by an open porous non-woven substrate.

The doctor blade casting method is a well-established method for this purpose [37]. With a gap size of usually around 150–200 µm the casting solution is flattened to a homogenous wet film. Further transportation gives the microphase separated or microphase separating BCP chains time to rearrange in the wet film and react to solvent evaporation induced gradients of concentration or temperature, before freezing the structure by a quick solvent non-solvent exchange, when it is immersed into a precipitation bath. Up to now the resulting block copolymer film thickness is limited to a minimum of ca. 11 µm using this method in our group [40].

The reduction of the substructure layer thickness and its density is the key to keep additional pressure losses low. The high polymer consumption per unit area has up to now limited the attractiveness manufacturing BCP membranes at larger scales, as these block copolymers are expensive. Further thickness reduction by doctor blade coating technique is limited, because the coating needs a certain polymer concentration usually in the range of 15–25 wt.% to get casting solutions of sufficiently high viscosity that do not penetrate into the non-woven support material too deeply (leading eventually to pore blocking) and to keep the selective top layer without defects (i.e., no dewetting). A successful coating of ≤10 µm thin PS-*b*-P4VP layer at the inner surface of a polyethersulfone hollow-fibre membrane was recently reported by our group with [41].

Printing techniques like offset printing, pad printing, gravure printing, screen printing flexographic printing which allow a patterning of the covered surface by transferring ink onto a substrate. In contrast, coating techniques that usually involve the pouring, painting, spraying, casting or smearing of a film, such as spin coating, doctor blading, dip-coating, painting, spray coating, slot-die coating, curtain coating or slide coating generally [42] do not offer this property. However, some of these techniques only work in a batch mode like the spin coating approach, which is prominent in the solar cell industry or DVD production and can homogenously cover 30 cm diameter samples on smooth substrates like silicon wafers. Spin coating has also been used in various studies on BCP self-assembly in thin films [43,44], but it cannot be transferred to a roll-to-roll (R2R) process for larger areas in contrast to e.g., profile roller coating or doctor blade casting.

R2R offers the possibility to include further production steps like heating, UV-light curing or drying, as necessary for dense membranes for e.g., gas separation [45] or a solvent/non solvent exchange in a precipitation bath in the case of porous membranes. R2R with smooth roles of aluminium and polytetrafluorethylene was used to produce macroscopically aligned microphase separated block copolymer films from solution before [46,47].

To the best of our knowledge, we report for the first time the casting of highly diluted BCP solutions in large scale with a profile roller coater in a SNIPS process, leading to membranes with thin layers of PS-*b*-P4VP. We will present the casting method and discuss the morphological features of the block copolymer layer, such as the thickness, structural homogeneity, polymer amount per unit area, and pH-responsive water flux. Also, the suitability of different supports is investigated. Furthermore, we demonstrate that this method allows the investigation of the influence of changing evaporation time in a single sample.

## 2. Materials and Methods

### 2.1. Synthesis of PS-b-P4VP

Synthesis of the block copolymer PS-*b*-P4VP was carried out via sequential living anionic polymerization following a synthesis route according to Rangou et al. [38]. (Further information is given in the Appendix A).

### 2.2. Solvents for Membrane Casting and Membrane Supports

The casting solutions of low viscosity (see Table 1) require a suitable support material that prevents excessive casting solution penetration, is resistant toward the used solvents and gives enough physical stability to the thin BCP layer. Non-woven polyester that is usually used to cast BCP membranes with a gap doctor blade [16,48,49] was not suitable for this purpose, because of the too high mesh distances which prevent the formation of a film on top with the low viscous casting solutions. In this work a home-made polyacrylonitrile (PAN) support membrane was used (see Appendix B.1). Home-made polyvinylidene fluoride (PVDF) and polyvinylidene fluoride + 8 wt.% titanium dioxide (PVDF + TiO_2_ (8%)) membranes were also not suitable candidates (see Appendix B.2 and Appendix B.3).

### 2.3. Casting Solutions for Membrane Preparation

All block copolymer (BCP) casting solutions were stirred until they appeared only slightly turbid. The slight turbidity of the solutions with the selective solvent DOX is due to the formation of micelles [28]. However, no sedimentation was observed, even after longer times. The solutions were stored overnight before further use. The polymer concentration was usually set to 1 wt.% in pure 1,4-dioxane (DOX) (see Table 1).

An overview of all casting solutions is given in Table 1. Table 1 listing the used BCP, the solvent composition, its density and viscosity.

Generally, the dynamic viscosity increases with increasing molecular weight in a range from 2.318–4.253 mPa·s, also depending on the ratio of block A to block B. The density of all 1 wt.% casting solutions was in the range of the pure solvent 1,4-dioxane with 1.03 g/cm^3^ and independent of the polymers molecular weight or the ratio of both blocks.

### 2.4. Nomenclature

The block copolymers (BCP) were used in different solvent systems usually with 1 wt.% polymer concentration. The following nomenclature is employed:

BlockA_x_-*b*-BlockB_y_^z^_SolventA_m_SolventB_n_^p^

where BlockA is the first block of the BCP, in this study usually polystyrene (PS), and BlockB is the second block, in this study poly(4-vinylpyridine) (P4VP). The subscripts x and y represent the weight ratio of each block in wt.%, whilst the superscript z is the total molecular weight in kg/mol. The solvent composition follows, where the subscripts m and n again describe the weight ratio in wt.% and p describes the percentage of block copolymer in wt.% within the given solvent system. In this work, mainly pure 1,4-dioxane (DOX) was used. The evaporation time before immersion into a precipitation bath can optionally form the last part of the designation.

Example: PS_83_-*b*-P4VP_17_^88k^_DMF_40_THF_60_^1^_15s

PS-*b*-P4VP with a polymer ratio of 83:17 and a molecular weight of 88,000 g/mol was dissolved in a mixture of DMF and THF with a ratio 40:60 at a concentration of 1 wt.%. The membrane cast out of this solution had an evaporation time of 15 s.

### 2.5. Viscosity and Density Measurements

The densities and viscosities of the used polymer solutions were measured with a DMA 4100 M (Anton Paar, Graz, Austria) density meter. The measurements were carried out with a 1.59 mm 1.4125-steel ball of 7.66 g/cm^3^ density in a 1.59 mm glass capillary at 20 °C.

### 2.6. Roller Coating

The casting process itself was carried out with a profile doctor blade (Zehntner GmbH, Sissach, Switzerland) of 0.32 m application breadth, 1.5 cm in diameter and with a weight of 359 g. It was mounted into two smooth-running ball bearings, 35 g each, which makes it a profile roller coater. The profile pattern was formed as described in the following section.

**Figure 1:**
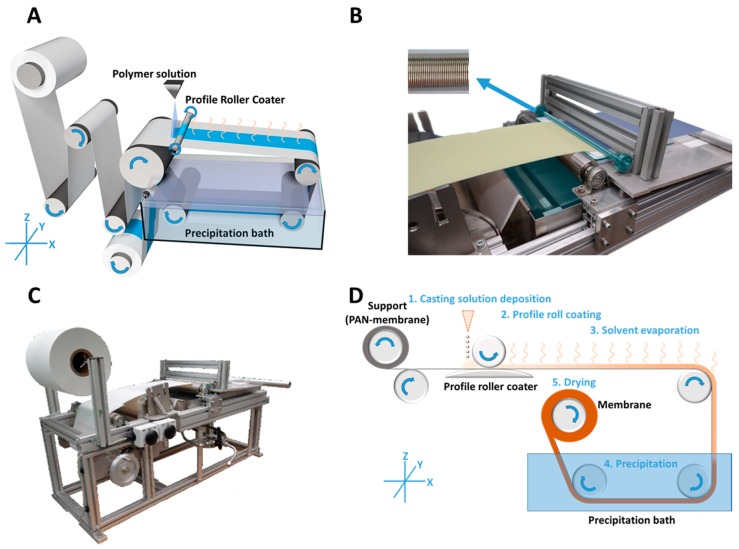
Casting process of roller coated membranes. Schematic drawing (**A**) image of the roller coater (**B**) and the in-house casting machine (**C**) for the casting process (**D**) of profile roller coated block copolymer (BCP) membranes. The casting process (**D**) is drawn in a simplified way with all relevant steps.

U-shaped milled rings into a rod are radially and periodically arranged, with a distance of 0.550 mm (Figure 1B; top-left) leading to a theoretical wet film thickness of 50 µm after the wave crests and troughs have converged to a uniform film.

The casting of the profile roller coated PS-*b*-P4VP membranes (Figure 1A) was carried out by continuous deposition of the casting solution (1) onto a moving PAN support membrane in front of the profile roller coater; the solution was immediately flattened to a homogenous wet film (2); the following solvent evaporation step gave the microphase separating system time to self-assemble (3) before immersion into a precipitation bath for quick solvent/non-solvent exchange, which froze the structure (4); in a last step, the resulting membrane was dried (5) (Figure 1D).

Further details:

A smooth glass plate below the support was employed to induce counter pressure. In order to compensate small thickness variations of the PAN support membrane, the ball bearings were movable in Z-axis (Figure 1). Hence the roller coater could move up or down with changing support thickness. The contact pressure was given by the operating weight of the profile coater and its two bearings with a mass of 429 g.

The volumetric flow rate of the low viscous casting solution was continuously adjusted to the consumption of the casting process itself, in order to prevent strong leakage at the edges or a lack of solution before the roller coater. A 5 mL syringe was used manually for this purpose, but this can easily be carried out with a volumetric flow pump or a spraying nozzle as well.

### 2.7. Film Thickness Gauge

For film thickness measurements of BCP-films cast on a PAN membrane, a magnetic inductive measurement device (ISO2178) DeltaScope^®^ FMP10 (Fischer GmbH, Sindelfingen, Germany) was used. Then, 100 data points were taken randomly on different positions of the sample.

### 2.8. Gravimetrical Polymer Consumption Measurements

The polymer consumption per unit area was determined (a) by measuring the required quantity of casting solution per unit gravimetrically with a Quintix 224-1S laboratory balance (Sartorius AG, Göttingen, Germany) and additionally (b) by the mass difference before and after coating with a XP105 analytical balance (Mettler Toledo, Greifensee, Switzerland). The Software ImageJ 1.51w (by Wayne Rasband) was used to determine the coated surface area by image analysis. All samples were dried under reduced pressure at 60 °C for at least 24 h before further investigations.

### 2.9. Photo Imaging under UV Light and Daylight Condition

An 8°W UV-lamp (Camag, Berlin, Germany) was used at a wavelength of 254 nm for imaging under UV light conditions. An EOS D60 camera (Canon, Krefeld, Germany) was used for imaging in manual mode with the settings f-number F5.6, ISO1000 and shutter speed 5 s, in dark room conditions.

### 2.10. Scanning Electron Microscopy (SEM)

A Merlin scanning electron microscope (Carl ZEISS, Oberkochen, Germany) was used for imaging of membrane samples at voltages between 3 kV up to 50 kV. The samples were sputter coated with 2.0 nm of a platinum layer to avoid charging. Cross-sections were prepared by dipping the membranes into *iso*-propanol, freezing in liquid nitrogen and subsequent cracking. The average pore size distribution and the average porosity were determined by analysing SEM-images using the software IMS (Imagic Bildverarbeitung AG, Glattbrugg, Switzerland).

### 2.11. Water Permeance Measurements

Pure water permeance measurements were carried out in dead-end mode using an in-house-device. The effective membrane area was 1.77 cm^2^. The transmembrane pressure (TMP) was detected by two LEO3 pressure gauges (Keller AG, Winterthur, Switzerland). Measurements were carried out at room temperature and at 1 bar TMP. The mass of the permeate was measured gravimetrically every minute. The first 15 min of measurement were excluded from calculations to give the membrane flux time to stabilise. We used demineralised pure water provided by a LaboStar^TM^ UV2 (Siemens; Berlin, Germany) with an electrical conductivity of ≈0.055 μS·cm^−1^. The permeance (*L*) is calculated by normalizing the flux to the TMP.

(1) $$L=\frac{\Delta V}{A\;\Delta t\;\Delta p}$$

Δ*V* is the volume of collected water and Δ*t* the time between two measurement points; *A* is the membrane’s top surface area and Δ*p* is the transmembrane pressure.

### 2.12. pH-Responsive Measurements

Solutions of 1 L pure water and 0.5 g sodium chloride (Merck kGaA; Darmstadt, Germany) were adjusted with hydrochloric acid (Sigma Aldrich, St. Louis, MO, USA) and sodium hydroxide (Sigma Aldrich) to pH = 3 and pH = 5, respectively, to test the pH responsive behaviour of the samples with regard to their permeance in a 200 mL Millipore testing cell (Merck kGaA; Darmstadt, Germany). The sample surface area was 1.77 cm². Starting with the solution with pH = 5 at 2 bar TMP, the first 15 min from permeance calculations were excluded to give the membrane time to equilibrate and then the mass change over the following 5 min was measured gravimetrically. The solution was then exchanged, the bottle was washed twice with the exchange solution (pH = 3) and this alternating procedure was carried out four times.

### 2.13. Retention Measurements

Solutions of 0.02 wt.% poly(ethylene glycol) (PEG) in H_2_O were used to perform retention measurements. The Mws of the used PEGs were 53, 106, 187 and 220 kDa. First, the pure water permeance was measured for 3 h at 2 bar TMP in a Millipore cell (Merck kGaA, Darmstadt, Germany), followed by substitution of the water with 100 mL PEG-solution. After 5 min stirring without applied pressure, a feed sample was taken. Subsequently, 30 mL of permeate were collected at 2 bar TMP and discarded in order to ensure equilibration and another 4 mL were collected afterwards as permeate sample.

The calculation of retention *R* is carried out using the following equation:

(2) $$R=1-\frac{c_{p}}{c_{f}}$$

The feed concentration *c_f_* and the permeate concentration *c_p_* of the samples were determined by gel permeation chromatography (GPC) at 35 °C in bidistilled water with 0.5 g/L sodium azide using PSS acrylate copolymer SUPREMA Pre, 100 Å and 3000 Å columns (particle size 10 µm), at a flow rate of 0.5 mL·min^−1^ (VWR—Hitachi 2130 pump). A Waters 410 index detector with a PEG calibration was used.

## 3. Results and Discussion

With the aim to reduce the consumption of block copolymer per unit area, in this work highly diluted casting solutions were used in contrast to conventional gap doctor blade cast membranes. The coating technique was successfully changed to a profile roller coating approach (see Section 2.6), which allowed for an additional reduction of the wet film thickness (WFT). The combination of both lead to thin porous PS-*b*-P4VP layers in the range of 0.5–3 µm after precipitation and drying, on top of a PAN support membrane. The savings of BCP per unit area easily exceed 95%. Additionally, a method to quickly investigate the influence of a changing evaporation time for the membranes using one sample only is presented. The support properties and the film homogeneity will also be discussed. Furthermore, the retention and pore size distribution of a PS_83_-*b*-P4VP_17__DOX_100_^1^ membrane and the PAN support will be considered.

### 3.1. Mass Per Unit Area

The great potential of the roller coating method becomes apparent at a closer examination of the polymer consumption per unit area in g/cm^2^. For a first theoretical approximation, the covered surface area of a roller coated sample was determined as 766 cm^2^. A wet film thickness of 50 µm was assumed based on manufacturer information. This data allows for the determination of the theoretical polymer consumption when the concentration of the casting solution is known, which was 1 wt.% in this case. The exact wet film thickness is not measurable due to the porous substrate, which immediately soaks in part of the solvent. However, the gravimetric mass change of a sample membrane with respect to its pristine support and the additional simple measurement of the required quantity of casting solution will allow for a more realistic comparison to a gap doctor blade cast membrane from 15.5 wt.% of PS_76_-*b*-P4VP_24_^330k^ in a mixture of DMF/THF (40/60). The results are shown in Table 2.

The polymer consumption of theoretically 0.5 g/m^2^ per unit area of the roller coated PS_83_-*b*-P4VP_17__DOX_100_^1^ is only 1.7% in comparison to the theoretical value of 31 g/m^2^ of a doctor blade cast PS_76_-*b*-P4VP_24_^330k^_DMF_40_THF_60_^15.5^ on a polyester non-woven with a gap size of 200 µm. A membrane with a relatively low concentration of 15.5 wt.% was chosen for comparison reasons. In practice, the consumption was even lower with 0.3%, namely 0.26 g/m^2^ for the roller coated instead of 81 g/m^2^ for the doctor blade cast membrane. The reason is the partial penetration into the non-woven support, which additionally increases the thickness of its wet film. The gravimetrically measured amount of the roller coated (RC) membrane is approximately half of its theoretical value, which is explainable because the 50 µm film thickness is a calculated value by the manufacturer, without considering rheological influences. In roller coating mode of operation not the whole casting solution is transferred to the support and parts remain within the profile. That is why the resulting film is thinner than a theoretical wet film of ~50 µm.

Because of the small gravimetric mass change of only 0.02002 g (±0.15 mg) for our ~766 cm^2^ (i.e., 0.26 g/m^2^) cast sample, the required quantity was also determined by examining the mass change of the casting solution over the production period which was 2.3065 g in total and hence 0.3 g/m^2^. The two values are in good agreement. The very small deviation between required quantity and gravimetric mass change also shows that losses due to the casting process are negligible. Small amounts remain on the roller coater itself or are applied to the edges of the membrane where the film gets thinner, i.e., a section that is not used as part of the homogeneous membrane and which was excluded from surface area calculations.

The applied mass of 2.3065 g also shows that the wet film must be thinner than the approximately 50 µm quoted by the manufacturer, since using this wet film thickness only an area of 444 cm^2^ instead of 766 cm^2^ would have been coated.

### 3.2. Coating and Film Homogeneity

The described coating technique (see Section 2.6) represents a straightforward way of coating thin BCP membranes in a SNIPS and R2R process. The setup could be improved, e.g., by replacing the glass plate with a counter roll equalising the applied force, by using a metric pump for controlled application of the casting solution or by transferring the casting solution indirectly from a reservoir with one or more rolls to the support (see Figure 1). However, even without these improvements it was possible to successfully coat thin BCP membranes over a large scale. The used PAN support showed a good wettability with the casting solution (additional information is given in Appendix G).

**Figure 2:**
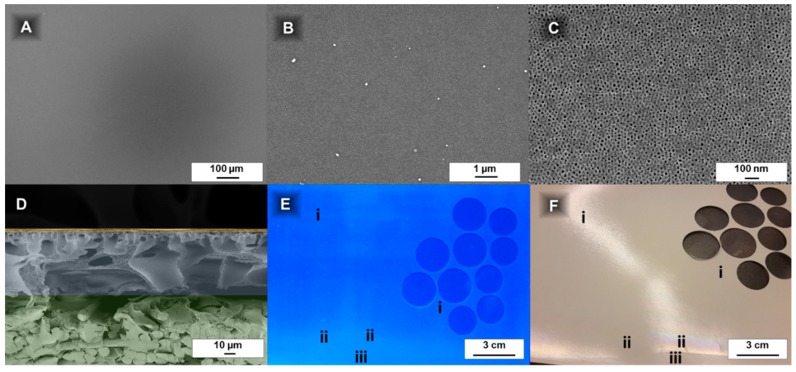
Film homogeneity quantification of a roller coated PS_83_-*b*-P4VP_17_^88k^_DOX_100_^1^ membrane: The scanning electron microscope (SEM) images show a homogenous coating in the range of 100 nm up to 1000 µm (**A**–**C**) without hints of defects; The cross-sectional view (**D**) indicates a homogenous layer thickness of PS_83_-*b*-P4VP_17_^88k^ (top; yellow) on top of the polyacrylonitrile (PAN) support membrane (PAN, middle; blue and non-woven, bottom; green) without penetration of the same; Under UV light 253 nm the PS-*b*-P4VP layer appears homogenous on a PAN membrane over a large scale and parts of the inhomogeneous coating could be easily made visible by this method (**E**); By observation of the reflections against a light source coloured areas of the PS-*b*-P4VP film indicate the beginning of the shrinking of the film thickness (ii) at the edges and that were excluded from further use (usually ~2 cm) (**F**). Locations designated by Roman numerals are explained in the text.

Because the film thickness is reduced to below 3 µm with this coating technique (Figure 2D and Figure 6; Appendix D), the film homogeneity evaluation becomes more difficult. SEM investigations were conducted to prove that the coating is uniform on a microscopic scale. Additional imaging under daylight and under UV-light conditions allows to evaluate the homogeneity on a larger scale.

#### 3.2.1. Investigation of Film Homogeneity by Daylight

For macroscopical homogeneity observation, an easy method to find deviations in the layer thickness is holding the sample against a light source and observe the reflection (Figure 2F). Areas with a changing colour indicate the PS-*b*-P4VP coating becoming very thin (e.g., close to the membrane edges).

This may sound trivial, but the porous support, its waviness and the thin PS-*b*-P4VP prevent simple and time efficient film thickness measurements (e.g., with a film thickness gauge device) (additional information is given in Appendix F). The determination of the thickness of a PS^83^-*b*-P4VP_17_^88k^_DOX_100_^1^ membrane with 15 s evaporation time cast on a PAN support membrane was attempted with a magnetic inductive film thickness gauge. A value of 168.8 µm (SD 9.43) was determined for the homogenous middle part of the membrane (Figure 2F-i). At the edge of the film (Figure 2F-ii) a value of 169.0 µm (SD 7.56) was measured and the pristine, uncoated PAN part (Figure 2F-iii) of the membrane had a thickness of 176.5 µm (SD 6.96). This comparison shows the deviations are too big to credibly calculate the PS-*b*-P4VP layer thickness by subtracting the PAN membrane thickness. Generally, the PS-*b*-P4VP or PtBS-*b*-P4VP layers appear smoother with a tendency to better reflect light in contrast to the PAN support (Figure 2F). However, it is difficult to draw a conclusion from a single image since changing light-reflections make the documentation difficult (see Figure 2F at position ii within and without of the light reflection). For this reason, a UV-light-method was employed to supplement the measurement technique described above.

#### 3.2.2. Investigation of Film Homogeneity by UV-Light

PAN showed fluorescence under UV-light with a wavelength of 254 nm [50], while the PS-*b*-P4VP layers remain black. With this method, a way to identify deviations in the PS-*b*-P4VP layer thickness was found. They appear as darker (for thicker layers) or brighter areas on a UV-light image. The shutter speed of the camera was increased to 5 s to get sufficient contrast for this purpose.

The films appeared homogenous over the whole casting area, except the 2–3 cm at the edges, like seen under day-light conditions (see Section 3.2.1; Figure 2F), where the PS-*b*-P4VP films become brighter and more and more closely resemble the uncoated purple colour of the pristine PAN, which is an indication for a thinner film. This method allows to straightforwardly evaluate the film quality in a qualitative way over a larger scale for fluorescing support materials.

#### 3.2.3. Investigation of Film Homogeneity by SEM

The top layer SEM images confirm a homogenous coating at a microscopic scale of up to 1 mm (Figure 2A–C) and the cross-sectional images reveal that there is nearly no deviation in layer thickness. Also microphase separation (Figure 2C) appears to be uniform over a large range.

### 3.3. Influence of the Evaporation Time on Membrane Morphology

The precipitation bath freezes the membrane in a non-equilibrated state. The continuous solvent evaporation induces gradients of polymer concentration, viscosity and temperature within the wet polymer film. The resulting membrane morphology is therefore strongly determined by the evaporation time prior to immersion of the film into the precipitation bath. In the following section a method is described that allows the facile investigation of the pore development with ongoing evaporation time using one sample only, instead of casting many single samples.

**Figure 3:**
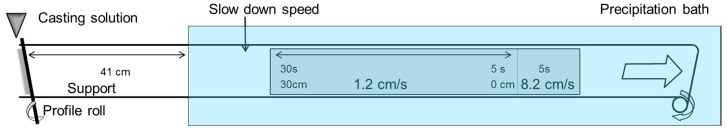
Casting process of gradient samples. The support moves with a constant speed of 8.2 cm/s. The casting solution is applied in front of a profile roll coater, which flattens the casting solution homogeneously to a wet film. After 41 cm distance, the casting solution comes into contact with the precipitation bath, i.e., after a period of 5 s. We call this zero position (0 cm). The speed is then reduced to 1.2 cm/s and therefore the evaporation time slightly increases along the sample up to 30 s at the 30 cm position.

As shown in Figure 3, such gradient sample membranes were cast with a speed of 8.2 cm/s and when they reached the precipitation bath after traveling 41 cm (i.e., 5 s of evaporation time), the velocity was decreased to 1.2 cm/s. This results in the evaporation times of the sample to continuously increase along the sample, in this case up to up to 30 s.

This method excludes deviations due to changing testing conditions (e.g., different room temperature, air humidity or air flow of the fume hood or changing concentration of the casting solution) which might affect experiments with individually produced samples.

**Figure 4:**
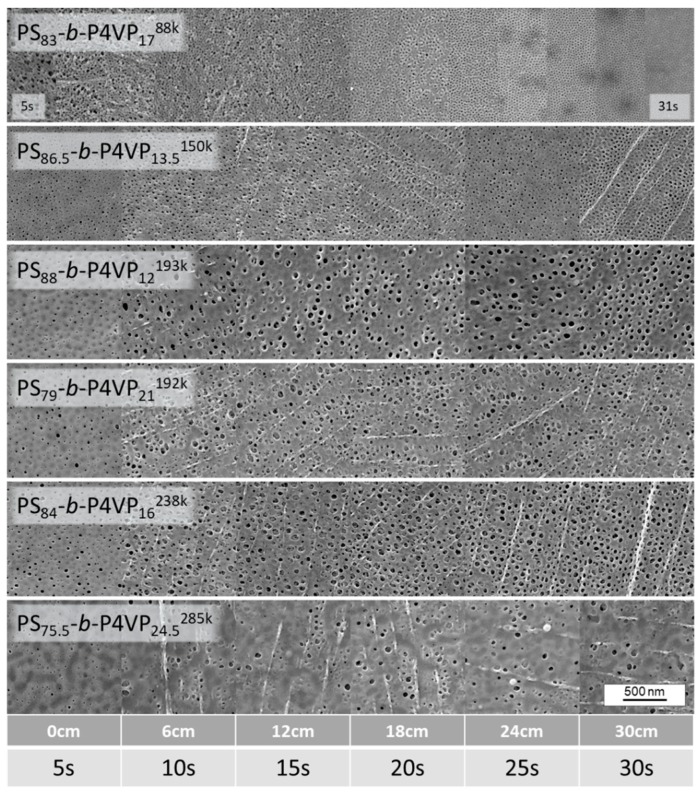
Gradient samples of different PS-*b*-P4VP membranes with a changing evaporation time along the sample. The casting speed was 8.2 cm/s and after 41 cm the velocity was reduced to 1.2 cm/s after application of the BCP-film. The left side (0 cm; 5 s) first met the precipitation bath. With this method, the changing pore morphology with ongoing evaporation was investigated in single samples to ensure constancy of all other influencing parameters like fluctuating fume hood strength, humidity or concentration deviations of the casting solutions.

Evaporation time gradient samples were prepared from all casting solutions listed in Table 1. As an example, the gradient sample of PS_83_-*b*-P4VP_17_^88k^_DOX_100_^1^ which shows an irregular structure after a short evaporation time of 5 s is presented. With increasing evaporation time, the pore structure becomes more regularly arranged with an optimum between 15–23 s. Afterwards the pores start collapsing again and become increasingly dense (Figure 4). According to SEM images, the average pore size after 15 s evaporation time for a PS_83_-*b*-P4VP_17_^88k^_DOX_100_^1^ was 12.5 nm (SD 3.3) (see Appendix C).

**Figure 5:**
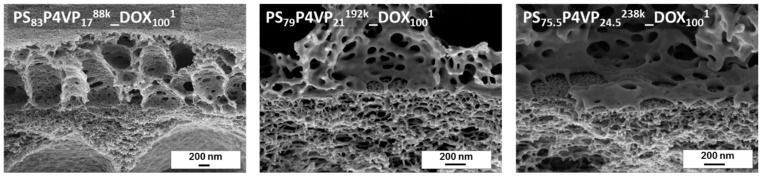
SEM cross-sectional images of the interface between PS-*b*-P4VP to PAN.

The tilted cross-sectional SEM images of PS-*b*-P4VP_DOX_100_^1^ membranes give information about the interface between the PS-*b*-P4VP-layer and the PAN support membrane. As can be seen, the BCP is not penetrating the PAN support. The PS-*b*-P4VP chains remained above the PAN layer, forming a highly open layer with large caverns (Figure 5).

**Figure 6:**
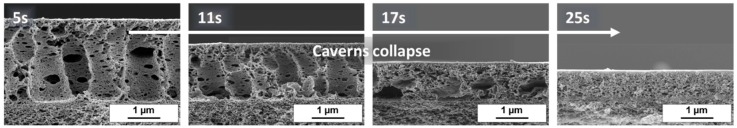
Cross-sectional image of a PS_83_-*b*-P4VP_17_^88k^ gradient sample at the position of 5 s, 11 s, 17 s and 23 s (left to right) evaporation time.

The cross-sectional view also reveals the cavern development with ongoing evaporation time (Figure 6). Whilst the spongy substructure is highly open with extremely large caverns after 5 s and a layer thickness approximately of 2.6 µm, it constantly shrinks with ongoing evaporation time with 1.8 µm, 1.2 µm and 0.9 µm after 11 s, 17 s and 23 s, respectively. The development of the membrane morphology nicely shows the dynamics of the system and the importance of freezing of the structure at the right moment.

The higher molecular weight PS-*b*-P4VP block copolymers also showed similar thin layer thicknesses. However, their pore morphology requires further improvement by tuning of the solvent composition. SEM cross sectional images are available in the Appendix D.

### 3.4. pH-Responsive Measurement

**Figure 7:**
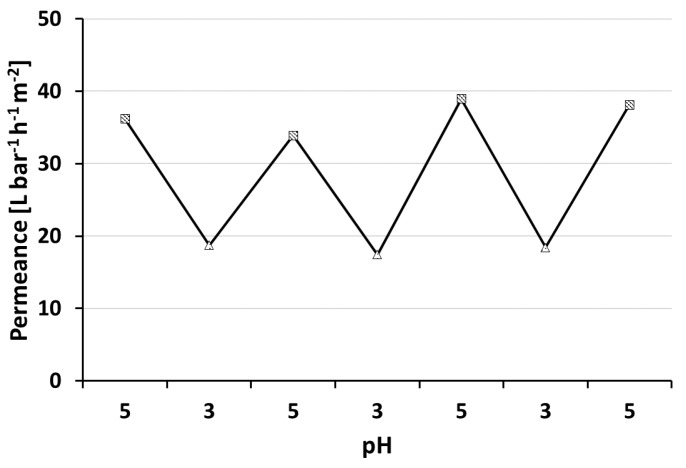
pH-responsive behaviour of a thin PS_83_-*b*-P4VP_17__DOX_100_^1^_15s. BCP membrane on a PAN support with four test cycles at pH = 3 (triangle) and pH = 5 (square) respectively. The transmembrane pressure was set to 2 bar at room temperature.

PS-*b*-P4VP membranes show the ability to respond to changing pH values of the feed solution (Figure 7). Lowering the pH value leads to protonation of the nitrogen’s free electron pair of the pore forming P4VP block. The repulsion of the positive charges in turn leads to a swelling of the polymer chains and to an effective pore size reduction in consequence. Hence a permeance decline is observed at lower pH-values. Changing the pH value between pH = 5 and pH = 3 confirmed that the pH-responsive, switchable nature for thin layered, profile roller coated PS-*b*-P4VP membranes is preserved. This process could be reproduced several times. Similar observations were made by our group with spray cast membranes prepared using the same polymer solutions but a PVDF support membrane [40]. However, generally higher water permeances of 400 L·bar^−1^·h^−1^·m^−2^ at pH = 5.1 were observed. The PVDF support was tested for roller coating application, but it was found to be not suitable in this case due to strong penetration that lead to defects (see Appendix B.2).

For both preparation methods, the supporting membrane was partly blocked by penetration of the BCP casting solution. We assume this blocked area might be higher in our case on a PAN support compared to the air brush spray cast samples on PVDF. However, this approach is designed for a scalable R2R-process, while the airbrush approach leads to circular samples of approximately 3 cm diameter and is probably difficult to upscale.

### 3.5. Retention

**Figure 8:**
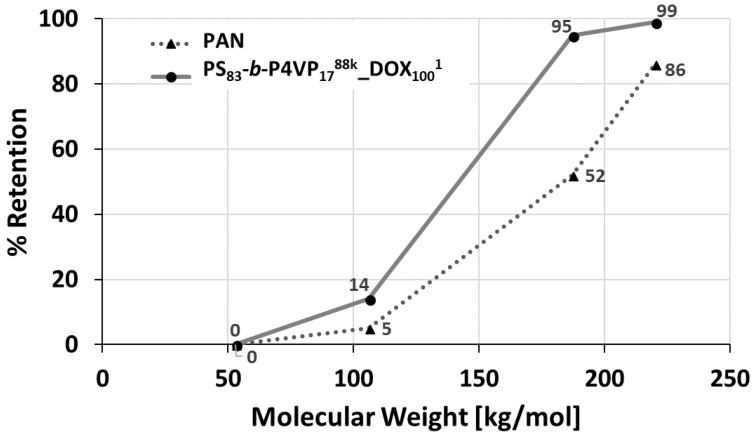
Retention measurement of a PS_83_-*b*-P4VP_17_^88k^_DOX_100_^1^ (circle) on PAN membrane and the pristine PAN membrane (triangle). Aqueous poly(ethylene glycol) (PEG) solutions of 0.2 wt.% with a MW of 53, 106, 187 and 220 kDa were used. The transmembrane pressure (TMP) was set to 2.1 bar. The concentration was determined by gel permeation chromatography (GPC).

The retention was determined with solutions of PEGs with molecular weights between 53 up to 220 kDa. The retention increased from 5% up to 95% between 106 kDa PEG and 187 kDa PEG, whilst the PAN membrane only showed 52% retention for 187 kDa PEG. Hence, the molecular weight cut-off (MWCO) >90% can be assumed in the range of 106–187 kDa (Figure 8).

The relatively sharp cut-off curve of the PS_83_-*b*-P4VP_17_^88k^_DOX_100_^1^ membrane is a result of its isoporous nature with a narrow pore size distribution observed on the SEM image (Figure 2C and Figure 4). At lower pH = 3, the retention of the PEG-106 kDa increased from 14% to 65% and for the PEG-55 kDa from 0% to 35%. This is a result of the pore shrinking of PS-*b*-P4VP membranes in acidic milieu as shown by pH-responsive water permeance measurements before (see Section 3.5).

### 3.6. Mechanical Properties

As it is difficult to determine mechanical properties of the thin coated membrane by simple methods such as tensile testing, the surface morphology of the membrane was investigated before and after bending and remained unaffected (Appendix E). Additionally, its stability was demonstrated by a stable pure water permeance, when changing the transmembrane pressure between 1 and 5 bar (Appendix E).

## 4. Conclusions

The BCP consumption per unit area was reduced by over 95% for BCP membrane manufacturing by implementation of a novel roller coating process. This was achieved by applying a thin layer of a BCP solution on top of a conventional PAN porous membrane. In comparison to doctor blade cast porous BCP membranes, the layer thicknesses were reduced to below 3 µm with this technique. Highly diluted solutions of 1 wt.% PS-*b*-P4VP in 1,4-dioxane have been used. Permeable PS-*b*-P4VP membranes with different molecular weights and compositions were obtained. A PS_83_-*b*-P4VP_17_^88k^ membrane was showing a molecular weight cut-off superior to that of the PAN support membrane and it showed pH responsive behaviour. Furthermore, a methodology was developed that allowed the investigation of the influence of different evaporation times on membrane formation using a specifically designed membrane production machine.

## Figures and Tables

**Table 1:** List of the used BCP solutions, their density, dynamic and kinematic viscosity.

Polymer	Density [g/cm³]	Dyn. Visc. [mPa·s]	Kin. Visc. [mm²/s]
1,4-dioxane (DOX_100_)	1.0336	1.340	1.296
PS_83_-*b*-P4VP_17_^88k^_DOX_100_^1^	1.0343	2.318	2.241
PS_86.5_-*b*-P4VP_13.5_^150k^_DOX_100_^1^	1.0342	3.989	3.857
PS_88_-*b*-P4VP_12_^193k^_DOX_100_^1^	1.0341	3.198	3.092
PS_79_-*b*-P4VP_21_^192k^_DOX_100_^1^	1.0341	2.881	2.785
PS_84_-*b*-P4VP_16_^238k^_DOX_100_^1^	1.0340	3.919	3.790
PS_75.5_-*b*-P4VP_24.5_^285k^_DOX_100_^1^	1.0343	4.253	4.112

**Table 2:** Polymer consumption per unit area of a roller coated PS_83_-*b*-P4VP_17_^88k^_DOX_100_^1^ in comparison to a gap doctor blade cast PS_76_-*b*-P4VP_24_^330k^_DMF_40_THF_60_^15.5^ membrane.

Property	Roller Coating	Doctor Blading
Gap size [µm]	(50 µm)	200 µm
Concentration [wt.%]	1 wt.%	15.5 wt.%
Theoretical [g/m²]	0.52	31
Gravimetric [g/m²]	0.26	81
Polymer reduction		-
Theoretical	98.3%	
Gravimetric	99.7%	
Required quantity [g/m²]	0.30	-
Polymer, solvent, conc.	PS_83_-*b*-P4VP_17_^88k^_DOX_100_^1^	PS_76_-*b*-P4VP_24_^330k^_DMF_40_THF_60_^15.5^
Support	PAN	Polyester

**Figure A1:**
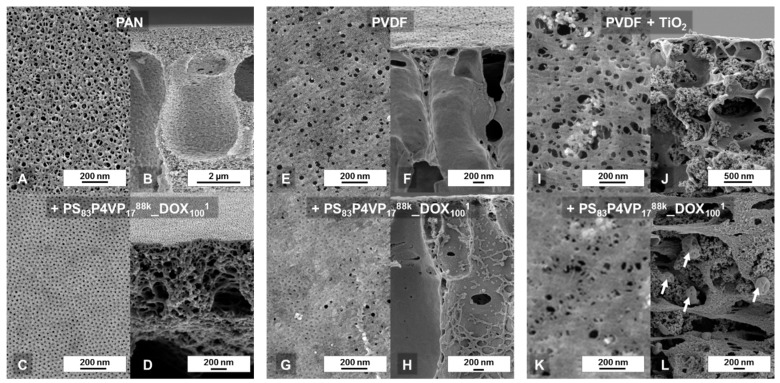
PAN (**A**–**D**), PVDF (**E**–**H**) and PVDF + TiO_2_ (8 wt.%) (**I**–**L**) as supporting membrane. The pristine top layer (**A**,**E**,**I**) and its cross-sectional view (B,F,J) and the same coated with PS_83_-*b*-P4VP_17__DOX_100_^1^ in top layer view (**C**,**G**,**K**) and cross-sectional view (**D**,**H**,**L**).

**Figure A2:**
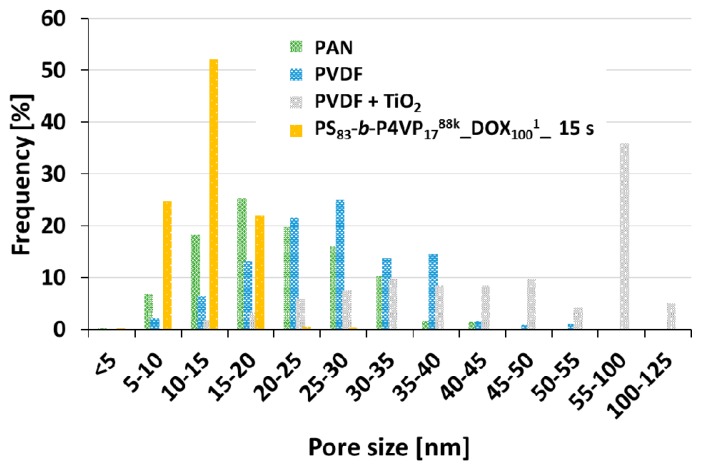
Pore size distribution measured by software on SEM images of different support membranes (PAN, PVDF and PVDF + TiO_2_ (8 wt.%)) and a PS_83_-*b*-P4VP_17_^88k^_DOX_100_^1^ on PAN membrane with 15 s evaporation time.

**Figure A3:**
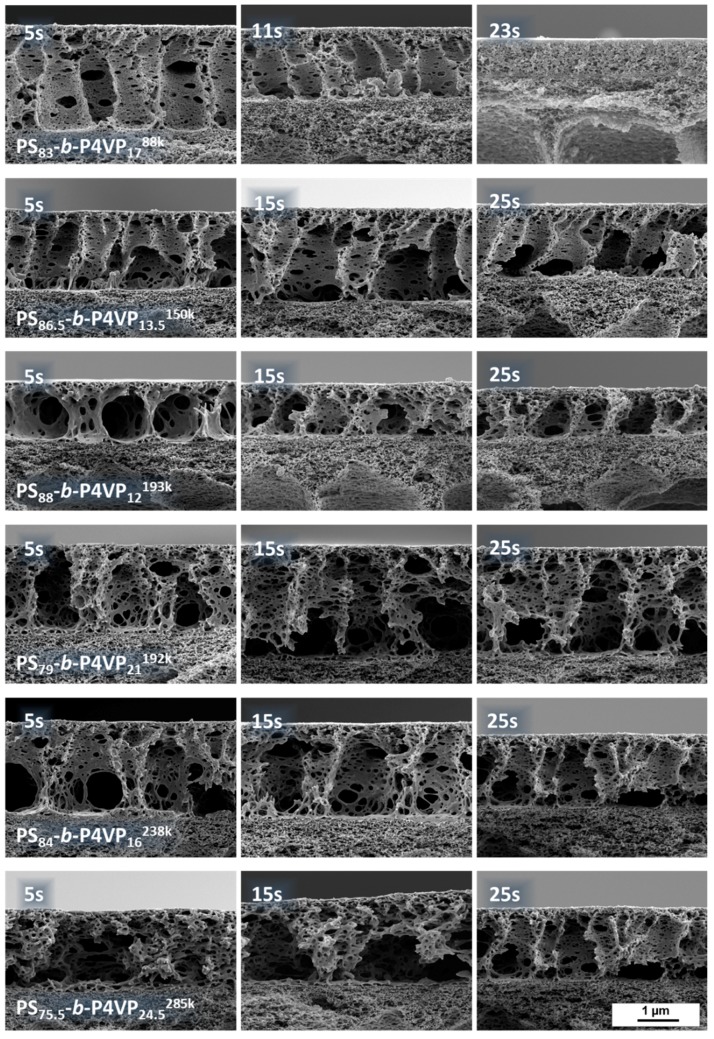
SEM cross-sectional view on PS-*b*-P4VP membranes of different casting solutions according to Table 1 with increasing evaporation time.

**Figure A4:**
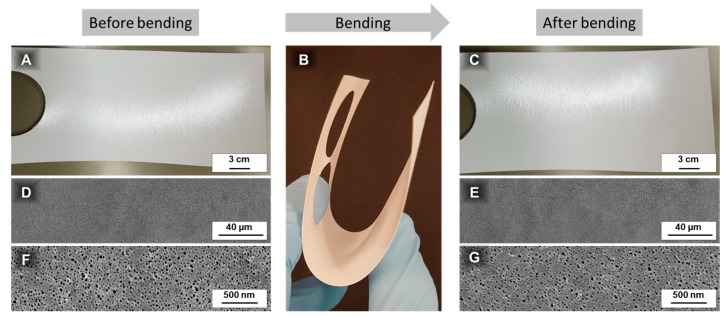
The physical strength of a roller coated PS_83_-*b*-P4VP_17__DOX_100_^1^_15s membrane was tested by bending a sample and comparing the surface before (**A**,**D**,**F**) and after (**C**,**E**,**G**) this test. Photographic image (**C**) and SEM-images (**E**,**G**) do not show any destruction of the sample after bending and remain unchanged.

**Figure A5:**
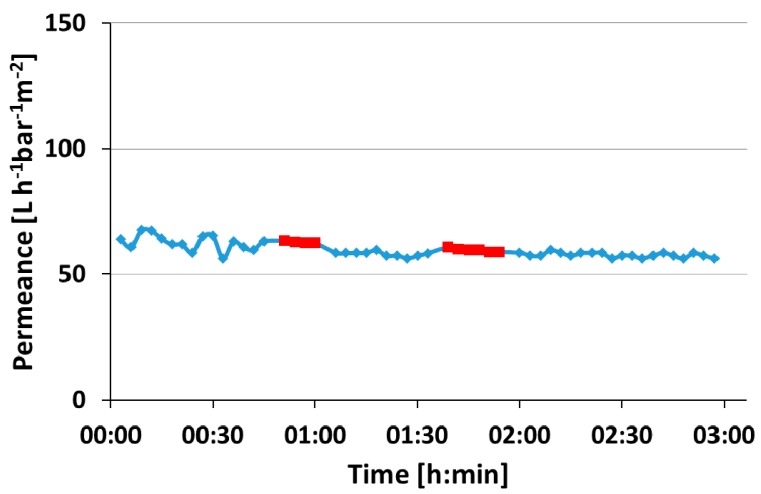
A pressure test of the roller coated PS_83_-*b*-P4VP_17__DOX_100_^1^_15s did not show a significant change of permeance when changing the transmembrane pressure from 1 bar (blue diamond) to 5 bar (red square) and backwards. The permeance stayed around 60 L·bar^−1^·h^−1^·m^−2^.

**Figure A6:**
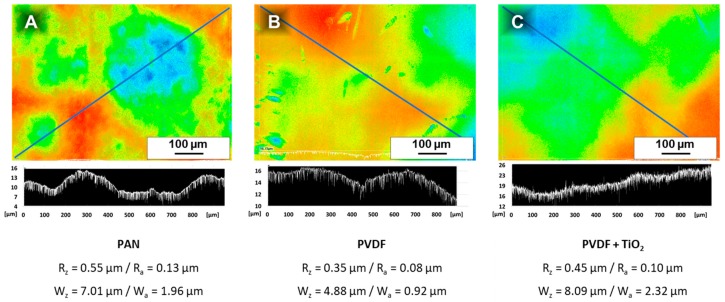
Laser scanning microscopic (LSM) images of the used support membranes PAN (**A**), PVDF (**B**) and PVDF + TiO_2_ (**C**). Calculations of roughness R_z_ and waviness W_z_ were performed over a distance of 950 μm (marked with a blue line). The profile along the selected part is shown below each LSM image.

**Figure A7:**
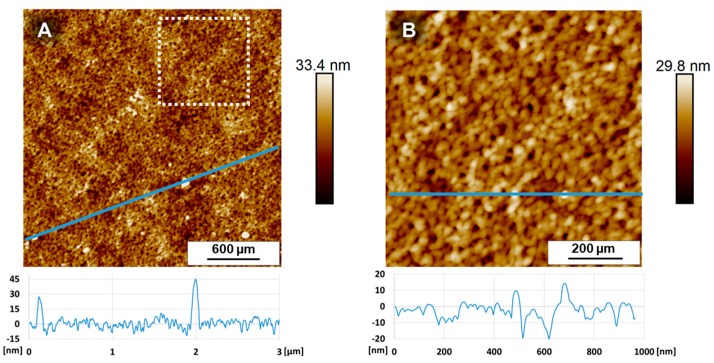
Atomic force microscopy (AFM) images of the PAN support membrane. (3 × 3) μm^2^ (**A**) and (1 × 1) μm^2^ (**B**) as enlarged part out of the first area (left image; white square). The roughness as depicted from the (3 × 3) μm^2^ image **(A)** is R_a_ ≈ 3.6 nm and from the 1 μm^2^ (**B**) is R_a_ ≈ 3.1 nm.

**Figure A8:**
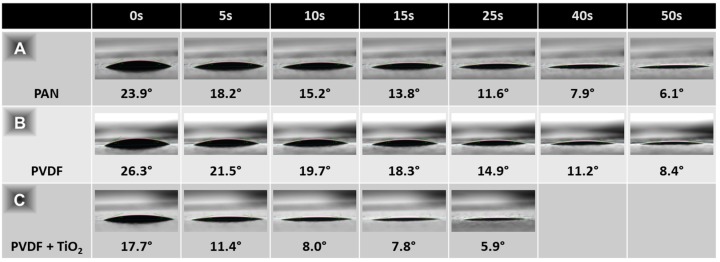
Dynamic contact angle measurements with 5 µL PS_83_-*b*-P4VP_17__DOX_100_^1^ casting solution on a PAN—(**A**), PVDF—(**B**) and PVDF + TiO_2_—(**C**) support membrane.

## Appendix A. Polymer Synthesis of PS-*b*-P4VP

Synthesis of the block copolymer PS-*b*-P4VP was carried out via sequential living anionic polymerization following a synthesis route according to Rangou et al. [38].

Styrene, 4-vinylpyridine (4VP), ethylaluminium dichloride (1 M in hexane), *sec*-buthyllithium (*sec*-BuLi) and dibutylmagnesium were purchased from Sigma-Aldrich. Tetrahydrofuran (THF) was purchased from Th.Geyer GmbH & Co. KG, Renningen, Germany.

THF was purified by successive distillation over potassium under argon atmosphere. Styrene was treated with dibutylmagnesium (MgBu_2_) and freshly distilled prior to use. 4VP was distilled under reduced pressure, stored over calcium hydride (CaH_2_) and distilled again after treating twice with ethylaluminium dichloride.

The sequential anionic polymerization of PS-*b*-P4VP was carried out in THF at −78 °C. The polymerization of styrene was initiated by *sec*-BuLi. After 2 h, 4VP was added and the solution was stirred for overnight. The polymerization was quenched with methanol/HCl. After removal of some THF under reduced pressure, precipitation of PS-*b*-P4VP was induced by dropwise addition of the polymer solution into a water bath. The polymer precipitated quantitatively and was filtered and dried in vacuum until constant weight.

The block copolymers fractional composition was determined via proton nuclear magnetic resonance (^1^H-NMR) spectroscopy using a Bruker AV-300 (Bruker BioSpin GmbH, Karlsruhe, Germany) at 300 MHz, in chloroform-*d* (CDCl_3_). The polydispersity and the molecular weight were measured at 50 °C by gel permeation chromatography (GPC) using a refractive-index detector Waters 2410 (Waters Corp, Milford, CT, USA) or ShodexRI-101 (ECOM, Prag, Czech Republic) and with dimethylacetamide (DMAc) as eluent. The system was calibrated with PS standards.

## Appendix B. Membrane Support

The casting solutions of low viscosity (see Section 2.3) require a suitable support material that prevents excessive casting solution penetration, is resistant toward the used solvents and gives enough physical stability to the thin BCP layer. Polyester non-woven that is usually used to cast BCP membranes with a gap doctor blade [16,48,49] was not suitable for this purpose, because the mesh distances were too high and would prevent a film from forming on top with the low viscosity casting solutions. In this work a home-made polyacrylonitrile (PAN) support membrane was used. Home-made polyvinylidene fluoride (PVDF) and polyvinylidene fluoride + 8 wt.% titanium dioxide (PVDF + TiO_2_ (8%)) membranes were also investigated as support membranes but were found to be unsuitable.

Hence, three different membranes have been tested as support for this purpose with different pore sizes:

- Polyacrylonitrile (PAN) membrane
- Polyvinylidene fluoride (PVDF) membrane
- Polyvinylidene fluoride + 8 wt.% titanium dioxide (PVDF + TiO_2_ (8%)) membrane

All membranes were stable against 1,4-dioxane (DOX) treatment without deformation.

### Appendix B.1. PAN

The polyacrylonitrile (PAN) membrane (Figure A1A) was cast with a 140 µm doctor blade from a PAN solution (8 wt.% in DMF). The overall PAN consumption was 12.1 g/m^2^ on a polyester non-woven for a 151 m × 0.7 m membrane. Its pure water permeance was 2322 (±73) L·bar^−1^·h^−1^·m^−2^, which was considered to be high enough for a suitable support. Whilst the entire PAN layer thickness was in the range of 60 µm, the upper layer from top to the beginning of the caverns shows a thickness of approximately 1 µm (Figure A1B). The biggest pore size as determined from SEM (Figure A1A) was 42 nm and the total porosity was 12.7% (see Appendix C).

No visible penetration of the BCP into this support was found (Figure A1D) and the BCP layer was found to completely cover the top over a large range (Figure 2A,B). The doctor blade casting technique of viscous solutions involves an inevitable, partial penetration of the PE-non-woven support. The physical entanglement with its fibres prevents delamination.

### Appendix B.2. PVDF

In comparison, an in-house PVDF membrane with a maximum pore size of 52 nm, a total porosity of 3.9% (see Appendix C) and a pure water permeance of 1957 (±24) L·bar^−1^·h^−1^·m^−2^ was also employed as a support structure. The SEM top view (Figure A1G) shows small areas of isoporous PS-*b*-P4VP films which are interrupted by zones of defects, which are assumed to correspond to the cross-sectional images, where PS-*b*-P4VP was found within the cavern system (Figure A1H).

### Appendix B.3. PVDF + TiO_2_ (8 wt.%)

A PVDF membrane with 8 wt.% TiO_2_ content had a maximum pore size of 124 nm, a total porosity of 9.5% (see Appendix C) and a pure water permeance of 8474 (±31) L·bar^−1^·h^−1^·m^−2^. Unfortunately, the desired high water permeance is accompanied by a too strong casting solution penetration. The completely dull surface of the cast membranes already gave a hint that the coating was not successful. The SEM images clarified that the casting solution did not stay on top forming a film (Figure A1K), but was nearly completely sucked into the support without film formation and only random structures of the BCP could be found in the SEM cross-sectional view (Figure A1L). Pore sizes of 124 nm are too high for applying the casting solutions of low viscosity used in this study.

## Appendix C. Pore Size Distribution by SEM

The software-based analysis of SEM images (Figure A2) of the used supporting membrane indicates a pore size distribution ranging from 4–43 nm (SD 7 nm) with a mean pore size diameter of 21 nm for the PAN membrane.

The PVDF membrane showed a slightly larger distribution between 5–52 nm (SD 9 nm). However, in cross-sectional view it was found that the casting solution had partially penetrated into this support type along the sample. For the PVDF + TiO_2_ (8 wt.%) membrane with a pore size distribution between 10–124 nm (SD 18 nm) and a mean pore size of 53 nm this effect was even more pronounced and the casting solution penetrated the support completely, which prevented the formation of a proper film on top. The maximum pore size without penetration should hence be smaller as 43 nm. With a pore size of 52 nm or higher the polymer chains of PS_83_-*b*-P4VP_17_^88k^ start penetrating the support leading to defects on top of the membrane.

As expected, the pore size distribution of the self-assembled PS_83_-*b*-P4VP_17_^88k^_DOX_100_^1^ BCP membrane with 15 s evaporation time, i.e., between 5–27 nm (SD 3 nm), showed a narrower pore size distribution in comparison to the afore mentioned conventional membranes.

A summary is given in Table A1.

**Table A1:** Summary of pore size distribution measured by software on SEM images of different support membranes (PAN, PVDF and PVDF + TiO_2_ (8 wt.%)) and a PS_83_-*b*-P4VP_17_^88k^_DOX_100_^1^ on PAN membrane with 15 s evaporation time.

Property	PAN	PVDF	PVDF + TiO_2_ (8%)	PS_83_-*b*-P4VP_17_^88k^
Min pore size	4.3 nm	5.3 nm	9.9 nm	4.6 nm
Max pore size	42.6 nm	52.4 nm	124.1 nm	26.5 nm
Average pore size	20.7 nm	26.8 nm	52.9 nm	12.5 nm
SD	6.9 nm	9.0 nm	17.8 nm	3.3 nm
Porosity	12.7%	3.9%	9.5%	2.1%

## Appendix E. Mechanical Properties

The mechanical strength of a PS_83_-*b*-P4VP_17__DOX_100_^1^_15s membrane was tested by manually bending a sample. No cracks were visually observed after this treatment. SEM images did not show any deformations or destruction of the membrane surface either (Figure A4).

In order to proof the mechanical stability of the membrane, the pure water permeance was measured for 35 min at 1 bar TMP, subsequently the TMP was raised to 5 bar for 15 min and afterwards reduced to 1 bar. After another 30 min measuring at 1 bar the procedure was repeated. The permeance remained constant before and after measuring at 5 bar TMP at around 60 L·bar^−1^·h^−1^·m^−2^, which implies that there is no indication for strong compaction, cracking or bursting of the membrane (Figure A5).

The membrane is therefore highly flexible and resists transmembrane pressures of 5 bar or more. In this study we used DOX as solvent and a PAN membrane as the support, but other solvents or solvent-systems like e.g., tetrahydrofuran (THF) and *N*,*N*-dimethylformamide (DMF) might help to improve the pore formation. The PAN support membrane is stable in DOX and tetrahydrofuran (THF), but can be dissolved by DMF. Solvent-systems containing these components are expected to generally work with this coating method. The PAN membrane should be replaced by a DMF-resistant material for higher DMF concentrations therefore or the concentration of DMF should be kept low.

## Appendix F. Roughness and Waviness

The roughness and waviness of the support membranes were determined by laser scanning microscopy (LSM) and by atomic force microscopy (AFM).

### Appendix F.1. Laser Scanning Microscopy (LSM)

A VK-X200 series laser scanning microscope (Keyence Deutschland GmbH, Neu-Isenburg, Germany) was used to illustrate the surface roughness and waviness of the used support membranes. 3 × 3 images (each with 2048 × 1536 pixel) performed with a 50× objective lens were automatically merged resulting in an image which covers an area of 800 × 583 μm^2^. The roughness and waviness were calculated by drawing a line of 950 μm length along the samples, which represents the peaks and valleys. The mean roughness depth R_z_, the arithmetic mean roughness value R_a_, the waviness W_z_ and the arithmetic mean waviness value W_a_ were calculated with cut-off wavelengths of λ_c_ = 0.08 mm and λ_s_ = 25 μm.

Laser scanning microscopy (LSM) images (Figure A6) of the PAN support show a waviness of W_z_ ≈ 7 μm and a roughness of R_z_ ≈ 0.6 μm. Therefore, the thin coating of PS-*b*-P4VP <3 μm on top cannot be detected by a film thickness gauge device and is part of the error of measurement.

The PVDF support membrane is smoother than the PAN with W_z_ ≈ 4.9 μm and R_z_ ≈ 0.4 μm, while the waviness of the PVDF + TiO_2_ support membrane is slightly higher at W_z_ ≈ 8.1 μm with R_z_ ≈ 0.5 μm.

While we expect the overall waviness over a distance of 0.95 mm to be trustworthy, because it is already visible by holding the sample against a light source, the short distance roughness is strongly dependent on the depicted cut-off wavelengths λ_s,_ which need to be set extraordinarily high to get similar results comparable to AFM images (see Appendix F.2). The AFM images of the PAN support over small distances of 3 μm × 3 μm show a much smoother surface roughness R_a_ ≈ 3.6 nm, which we evaluate to be more trustworthy due to matching observations made on SEM images (see Figure 2).

We believe this deviation probably occurs due to the challenging auto-focusing of the laser on the porous support membrane surface, which is not completely reliable. However, while the roughness is low, the waviness of the supports is already higher as the thickness of the PS-*b*-P4VP layer, therefore excluding the measurement with a film thickness gauge.

### Appendix F.2. Atomic Force Microscopy (AFM)

Atomic force microscopy (AFM) was carried out on a Multimode 8 (Bruker Corp., Billerica, MA, USA). The measurement was performed in tapping mode using a TAP150 tip (Bruker Corp., Billerica, MA, USA).

AFM images of the PAN support membrane with an area of (3 × 3) μm^2^ show a surface roughness of R_a_ ≈ 3.6 nm. A slightly lower value of R_a_ ≈ 3.1 nm was estimated, when depicting a (1 × 1) μm^2^ area out of the previous area and after measuring this part again with a higher resolution (Figure A7, right). The small deviation is explainable by small polymer pellets, which are randomly spread as white spots on this membrane surface. Their height was in a range of 30–45 nm.

## Appendix G. Dynamic Contact Angle Measurement

Dynamic contact angle measurements were performed on a DSC100 (Krüss GmbH, Hamburg, Germany) in combination with the software Drop Shape Analyser 1.8.4 (Krüss GmbH, Hamburg, Germany). For droplet deposition, the casting solution PS_17_-*b*-P4VP_83_^88k^_DOX_100_^1^ was used. A droplet of approximately 5 μL was deposited on the support membranes manually using a syringe with a screw. The contact angle was calculated by the software using “Tangent-1”-mode.

The PAN support shows good wettability with PS_83_-*b*-P4VP_17__DOX_100_^1^ casting solution in dynamic contact angle measurements. The initial contact angle, directly after depositing a 5 µL droplet, was 24° and decreased to 12° after 25 s.

The PDFV support had a slightly higher contact angle of 26° at the beginning with the coating solution and decreased to 15° after 25 s. The PVDF + TiO_2_ membrane showed the lowest initial contact angle of 18°, which decreased to 6° after 25 s (Figure A8).

The wettability and spreading behaviour of the used PAN support membrane is high enough for coating purposes, which is also the case for the PVDF and the PVDF + TiO_2_ membrane. Although the latter were not suitable for coating due to casting solution penetration.
